# Supplementary material for: Weak and Tunable Adhesion–Clutch Drives Rapid Cell Migration and Glioblastoma Invasion
Source: Adv Sci (Weinh). 2025 Aug 13;12(39):e02074. doi: 10.1002/advs.202502074 (PMC12533206; doi:10.1002/advs.202502074)
Supplement: Supplementary file 1 — Supporting Information [file ADVS-12-e02074-s002.docx]

**Supporting Information**

**Weak and Tunable** **Adhesion-Clutch Drives Rapid Cell Migration and Glioblastoma Invasion**

Kentarou Baba^1^, Ami Fukushi-Kumagai^1^, Megumi Morisaki^1^, Ryosuke Takeuchi^1^, Zhize Xiao^2^, Yoshikazu Nagashima^1^, Mizuki Sakai^1^, Yasuna Higashiguchi^1^, Hiroko Katsuno-Kambe^1^, Asako Katsuma^3^, Yoshihiro Ueda^4^, Yuji Kamioka^4^, Daisuke Kawauchi^2^, Tatsuo Kinashi^4^, Yonehiro Kanemura^3,5^, Naoyuki Inagaki^1,6,^*

^1^Division of Biological Science, Nara Institute of Science and Technology, Ikoma, Japan

^2^Department of Neuro-Oncology, Institute of Brain Science, Nagoya City University, Nagoya, Japan

^3^Division of Regenerative Medicine, Department of Biomedical Research and Innovation, Institute for Clinical Research, NHO Osaka National Hospital, Osaka, Japan

^4^Department of Molecular Genetics, Institute of Biomedical Science, Kansai Medical University, Hirakata, Japan

^5^Department of Neurosurgery, NHO Osaka National Hospital, Osaka, Japan

^6^Lead contact

*Correspondence: ninagaki@bs.naist.jp

**This file includes:**

・Supplementary Figures S1-S10

・Supplementary Table S1

・Supplementary Videos S1-S13 legends

**・**References

**
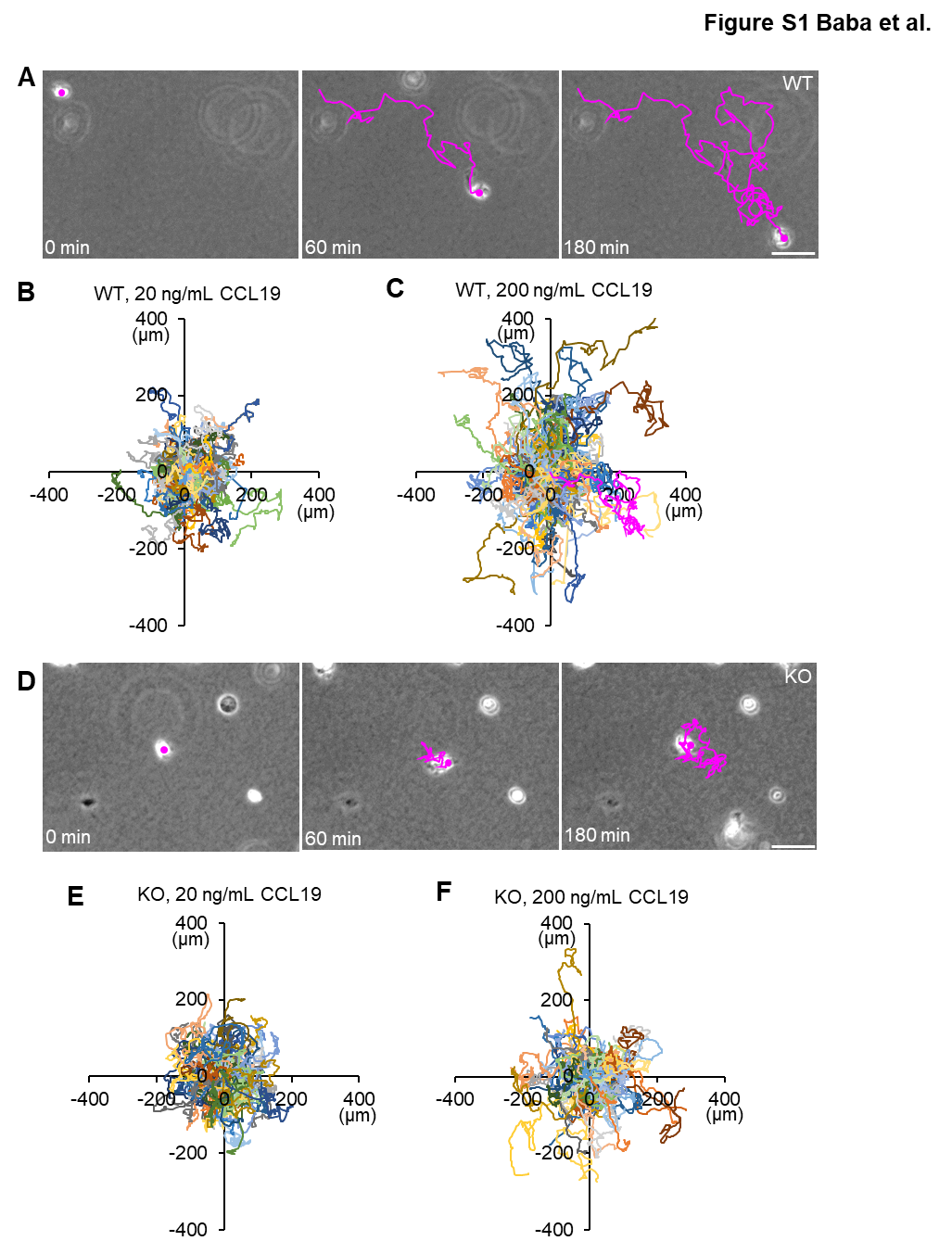
**

**Figure S1.** Shootin1b promotes dendritic cell migration under CCL19 signaling. A, D) Dendritic cells were cultured in a mixture of collagen gel and Matrigel. One hour after the bath application of 200 ng/mL CCL19, time-lapse phase-contrast/fluorescence images of WT (A) and shootin1b KO (D) dendritic cells were obtained. Nuclei were visualized by Hoechst to accurately trace the trajectories of cell migrations (see Video S2). The pictures show representative images from the time-lapse series taken every 1 min for 180 min. Scale bars: 50 µm. B, C, E, F) Trajectories of the migrations of WT (B, C) and shootin1b KO (E, F) dendritic cells in the presence of 20 ng/mL (B, E) and 200 ng/mL (C, F) CCL19. The initial cell positions are normalized at x = 0 µm and y = 0 µm.


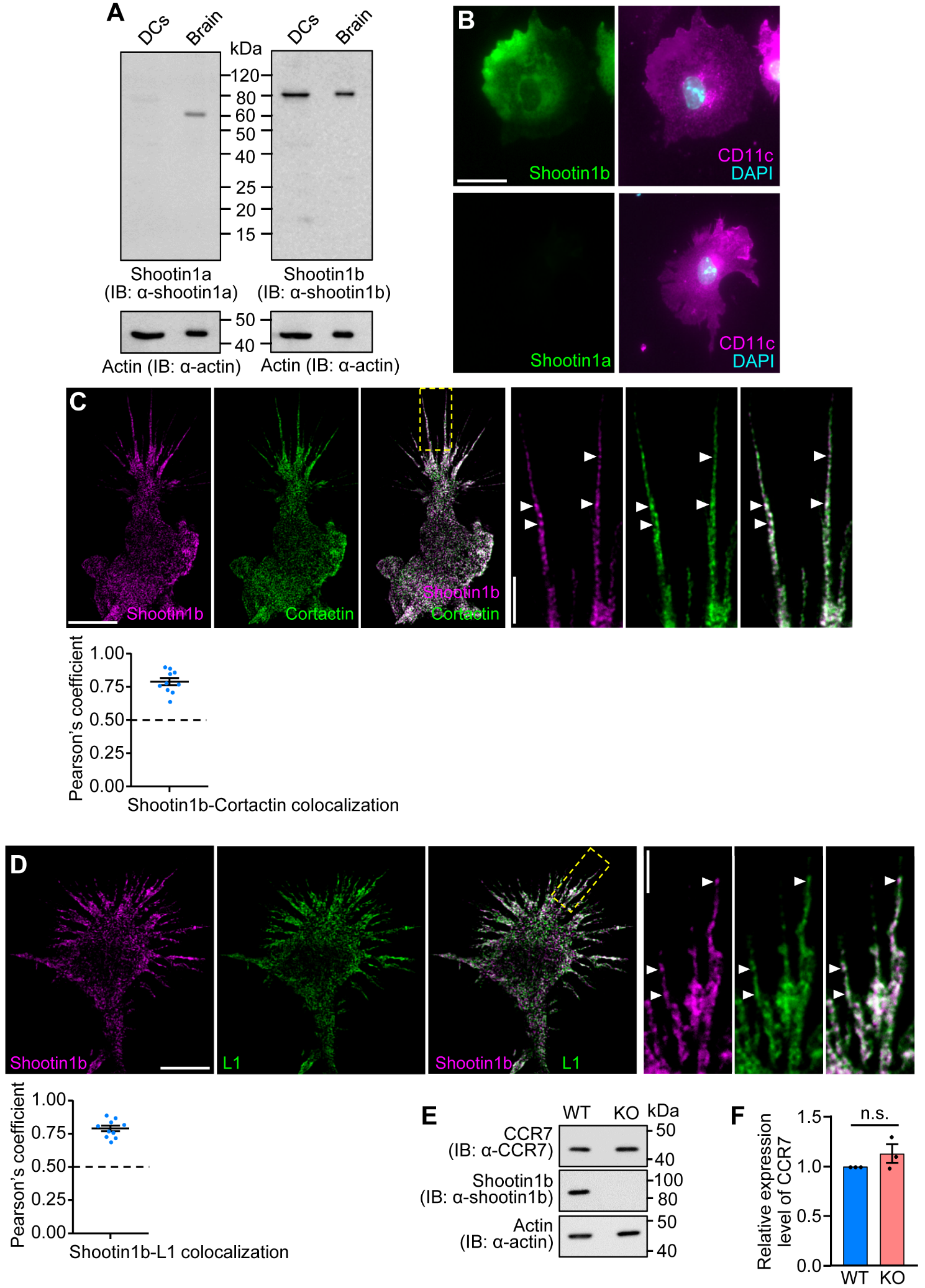


**Figure S2.** Expression and localization of shootin1b in dendritic cells. A) Immunoblot analysis of dendritic cells with anti-shootin1a and anti-shootin1b antibodies. Embryonic day 16.5 mouse brain lysate was used as positive control for the detection of shootin1a and shootin1b. Actin was used as a loading control. B) Fluorescence images of dendritic cells co-stained with anti-CD11c (dendritic cell marker) and anti-shootin1b or anti-shootin1a antibody. Cell nucleus was visualized by DAPI. Scale bar: 10 µm (in the inset, 2 µm). C, D) Fluorescence images of dendritic cells co-stained with anti-shootin1b antibody and anti-cortactin (C) or anti-L1 antibody (D). Enlarged views of the rectangular region are shown to the right. Arrowheads indicate shootin1b localization with cortactin (C) or L1 (D) in filopodia. The images were obtained by STED microscopy. Scale bar: 10 µm (in the inset, 2 µm). The lower graphs show the quantification of shootin1b-cortactin and shootin1b-L1 colocalization using Pearson’s coefficient correlation. The Pearson’s coefficient correlations at the leading edge (within 10 µm from the cell tip) from 10 cells (n = 10) exceeded 0.5 (0.78 ± 0.03 for shootin1b-cortactin and 0.79 ± 0.02 for shootin1b-L1), indicating the colocalization of shootin1b-cortactin and shootin1b-L1 at the leading edge of dendritic cells. E, F) Shootin1b KO does not affect the expression of the CCL19 receptor CCR7 in dendritic cells. Immunoblot analysis of WT and shootin1b KO dendritic cells with anti-CCR7 antibody (E). Actin was used as a loading control. Quantitative data for the CCR7 expression level in (F). Two-tailed unpaired Welch’s *t*-test was performed (n = 3 independent experiments). Data represent means ± SEM; ns, not significant.


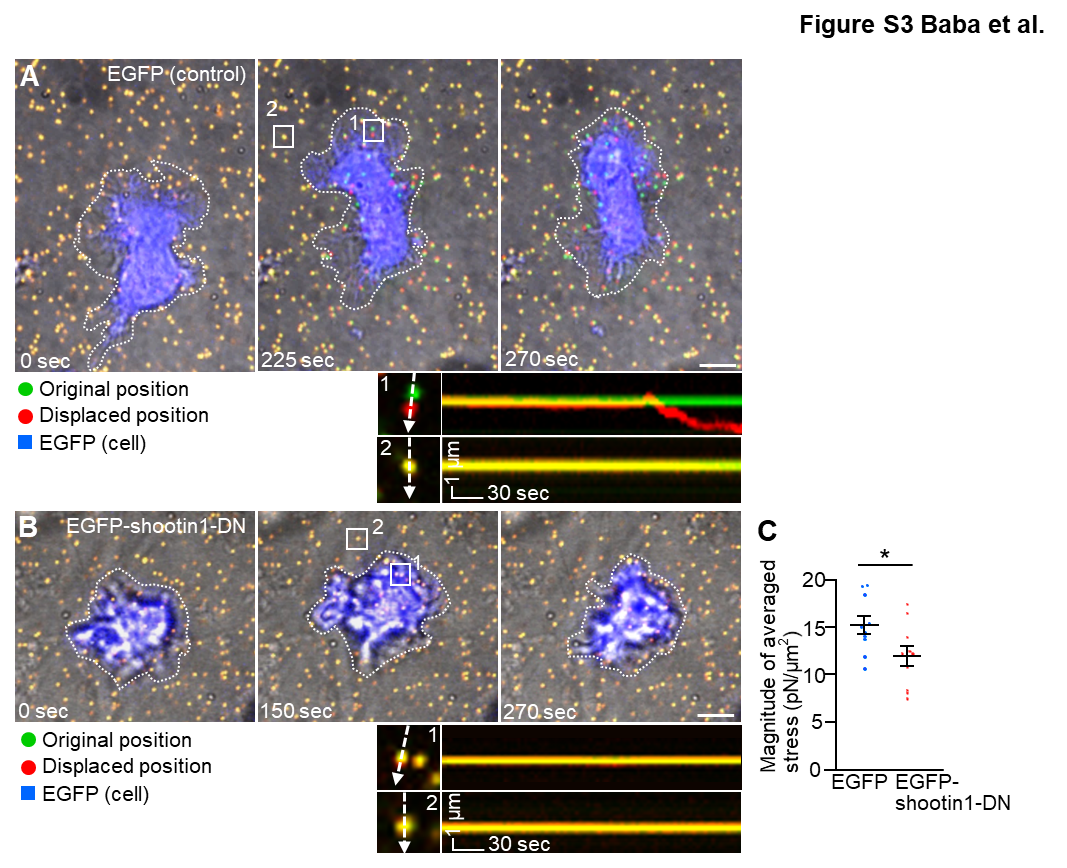


**Figure S3.** Shootin1b-L1 interaction mediates generation of weak forces by dendritic cells. A, B) Overlayed DIC and fluorescence images showing dendritic cells overexpressing EGFP (control) (A) or EGFP-shootin1-DN (B) migrating under the semi-3D condition in (Figure 1A) in the presence of 200 ng/mL CCL19. See Video S4. The pictures show representative images from the time-lapse series taken every 3 sec for 270 sec. The original and displaced positions of the beads in polyacrylamide gels are indicated by green and red colors, respectively. The cells were visualized by EGFP (blue color); dashed lines indicate the boundaries of the cells. The kymographs (panel below) along the axis of bead displacement (white dashed arrows) at indicated areas 1 and 2 show movement of beads recorded by every 3 sec. The bead in area 2 is a reference bead. Scale bar: 5 µm (in the inset, 1 µm). C) Analyses of magnitude of the traction force under the dendritic cells overexpressing EGFP or EGFP-shootin1-DN in (A, B). Two-tailed unpaired Student’s *t*-test (EGFP, n = 10 cells; EGFP-shootin1-DN, n = 10 cells). Data represent means ± SEM; *, p < 0.05.


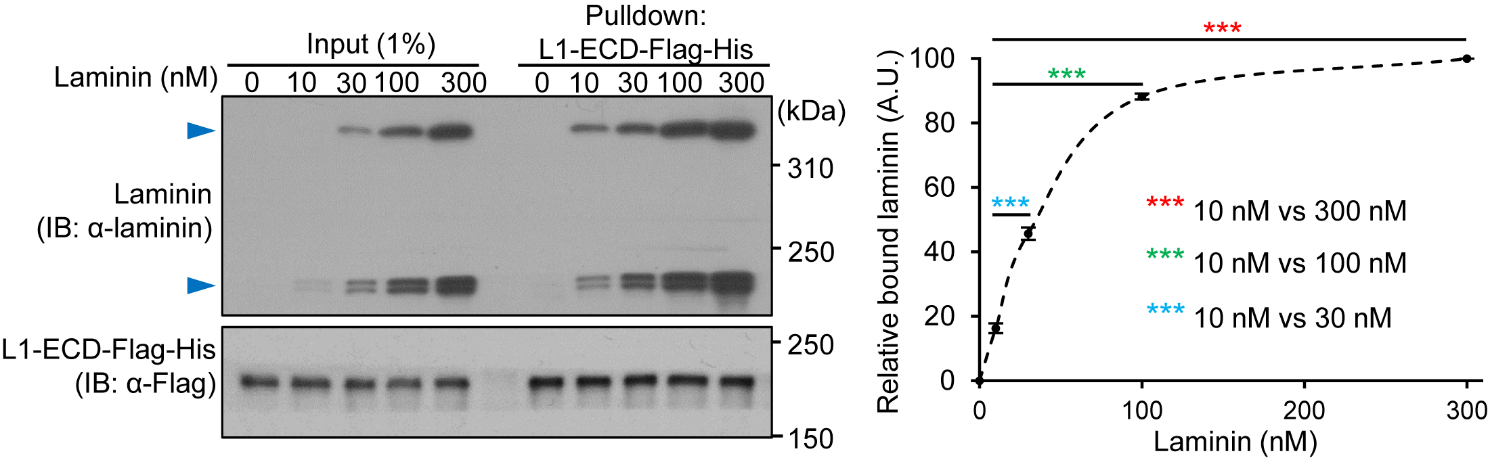


**Figure S4**. L1-ECD directly interacts with laminin. In vitro binding assay using purified laminin and purified L1-ECD (extracellular domain) with Flag-tag and His-tag at C-terminal region. L1-ECD-Flag-His proteins were expressed in HEK293T cells and purified by Ni- sepharose column from the supernatants of culture media without serum. L1-ECD-Flag-His was mixed with laminin at the increasing laminin concentration. The mix solution was incubated with Ni-NTA agarose beads. L1-ECD-Flag-His proteins were eluted by 200 mM imidazole in Tris-HCl buffer (pH 8.0). After SDS/PAGE, the eluate was immunoblotted with anti-laminin and anti-FLAG antibodies (Left). As reported previously[^1^](#_ENREF_1), laminin is composed of two bands at 220 kDa and 440 kDa (arrowheads). L1-ECD-bound laminin was then quantified at each laminin concentration (Right). One-way ANOVA with Turkey’s post hoc test was performed for multiple comparison of the data (n = 3 independent experiments). Data represent means ± SEM (n = 3). ***, p < 0.01


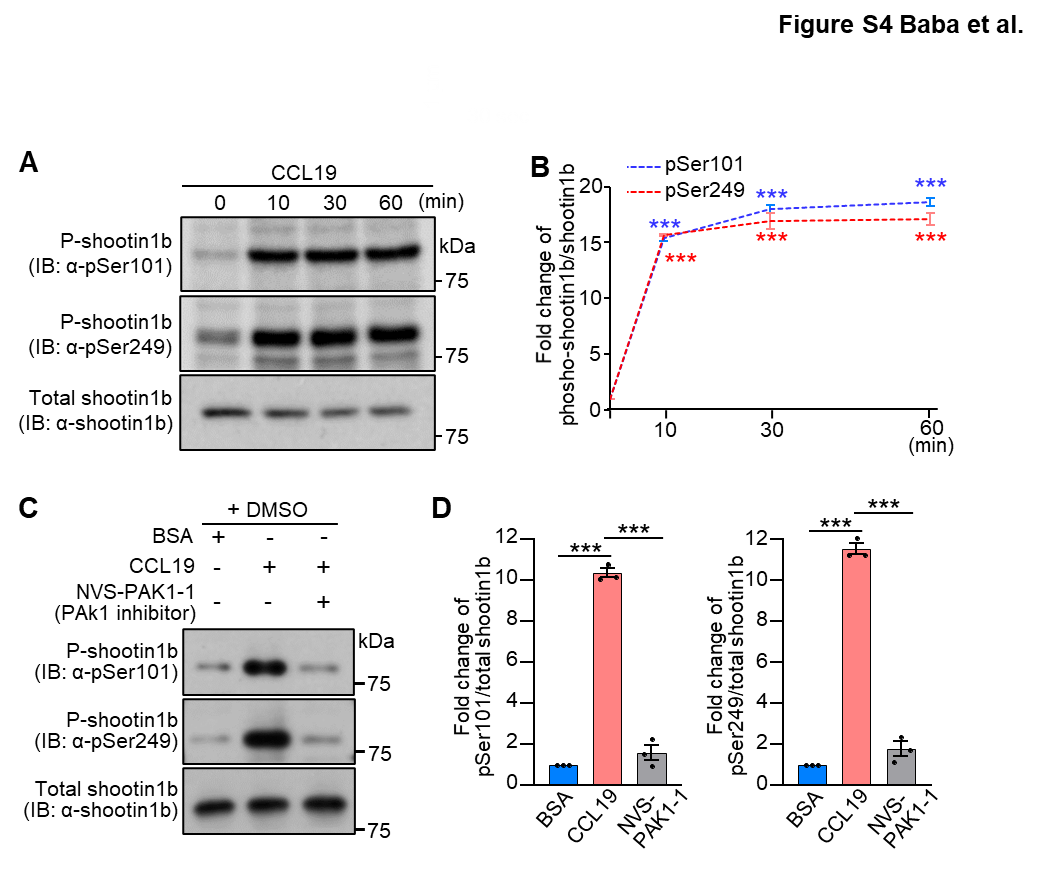


**Figure S5.** Pak1-mediated shootin1b phosphorylation under CCL19 signaling. A, B) Dendritic cells were treated with 200 ng/mL CCL19 for 0, 10, 30 or 60 min. Cell lysates were then analyzed by immunoblot with anti-pSer101 shootin1, anti-pSer249 shootin1 and anti-shootin1b antibodies (A). Quantitative data for phospho-shootin1b levels at 0, 10, 30 or 60 min are shown in (B). For multiple comparison, one-way ANOVA with Turkey’s post hoc test was performed (n = 3 independent experiments). C, D) Dendritic cells were treated with 200 ng/mL CCL19 or 0.1 % BSA (for control) for 30 min (C). To inhibit Pak1 function, 0.25 µM Pak1 inhibitor (NVS-PAK-1) was applied for 30 min before the CCL19 treatment. Cell lysates were then analyzed by immunoblot with anti-pSer101 shootin1, anti-pSer249 shootin1 and anti-shootin1b antibodies. Quantitative data for phospho-shootin1b levels are shown in (D). For multiple comparison, one-way ANOVA with Turkey’s post hoc test was performed (n = 3 independent experiments). Data represent means ± SEM; ***, p < 0.01.


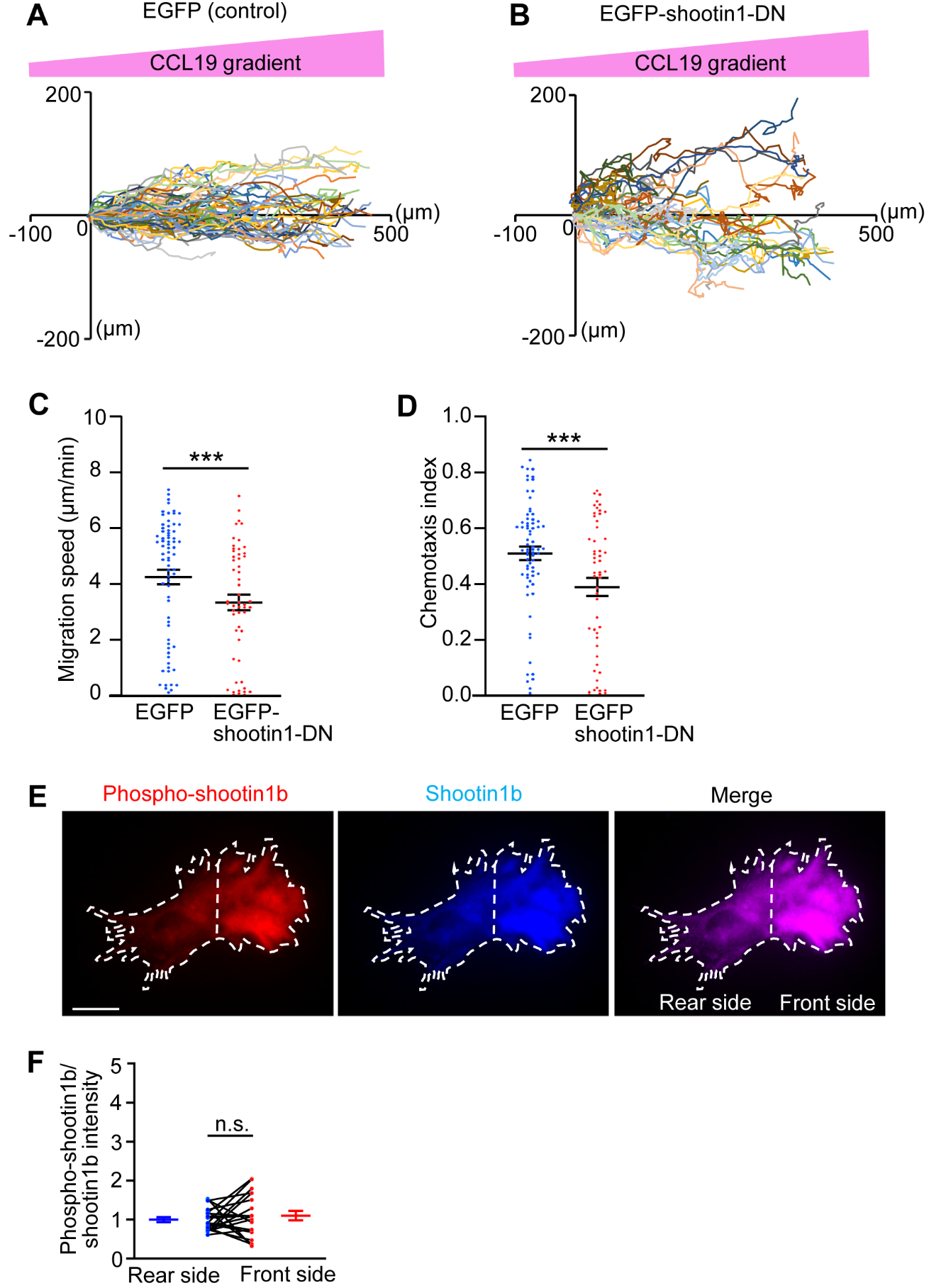


**Figure S6.** Shootin1b-L1 interaction mediates CCL19-induced dendritic cell chemotaxis. A, B) A gradient of CCL19 was applied to dendritic cells overexpressing EGFP (control) (A) or EGFP-shootin1-DN (B) cultured in a mixture of collagen gel and Matrigel (left panel, Figure 4A). The graphs depict the migration trajectories of individual dendritic cells overexpressing EGFP (A) or EGFP-shootin1-DN (B). The initial cell positions are normalized at x = 0 µm and y = 0 µm. See also Video S8. C, D) Analyses of migration speed (C) and chemotaxis index (D) for dendritic cells overexpressing EGFP (control) (A) and EGFP-shootin1-DN (B). Two-tailed Mann-Whitney *U*-test was performed (EGFP, n = 71 cells; EGFP-shootin1-DN, n = 54 cells). Data represent means ± SEM; ***, p < 0.01. E) Dendritic cells were transfected with myc-shootin1b to visualize shootin1b. After the stimulation by 200 ng/mL CCL19 bath application for 60 min, they were fixed and immunolabeled with anti-myc and anti-pSer249 shootin1 antibodies. Fluorescence images show the detected phosphorylated shootin1b and shootin1b in a dendritic cell. White dashed lines indicate the boundary of a dendritic cell and the center line that separates the front side and rear side. Scale bar: 10 μm. F) Quantitative data for shootin1b activation (phospho-shootin1b/shootin1b) in the front side and rear side of dendritic cells. Two-tailed paired Student’s *t*-test for phospho-shootin1b/total shootin1b between the front side and rear side (n = 20 cells). Data represent means ± SEM; ns, not significant.


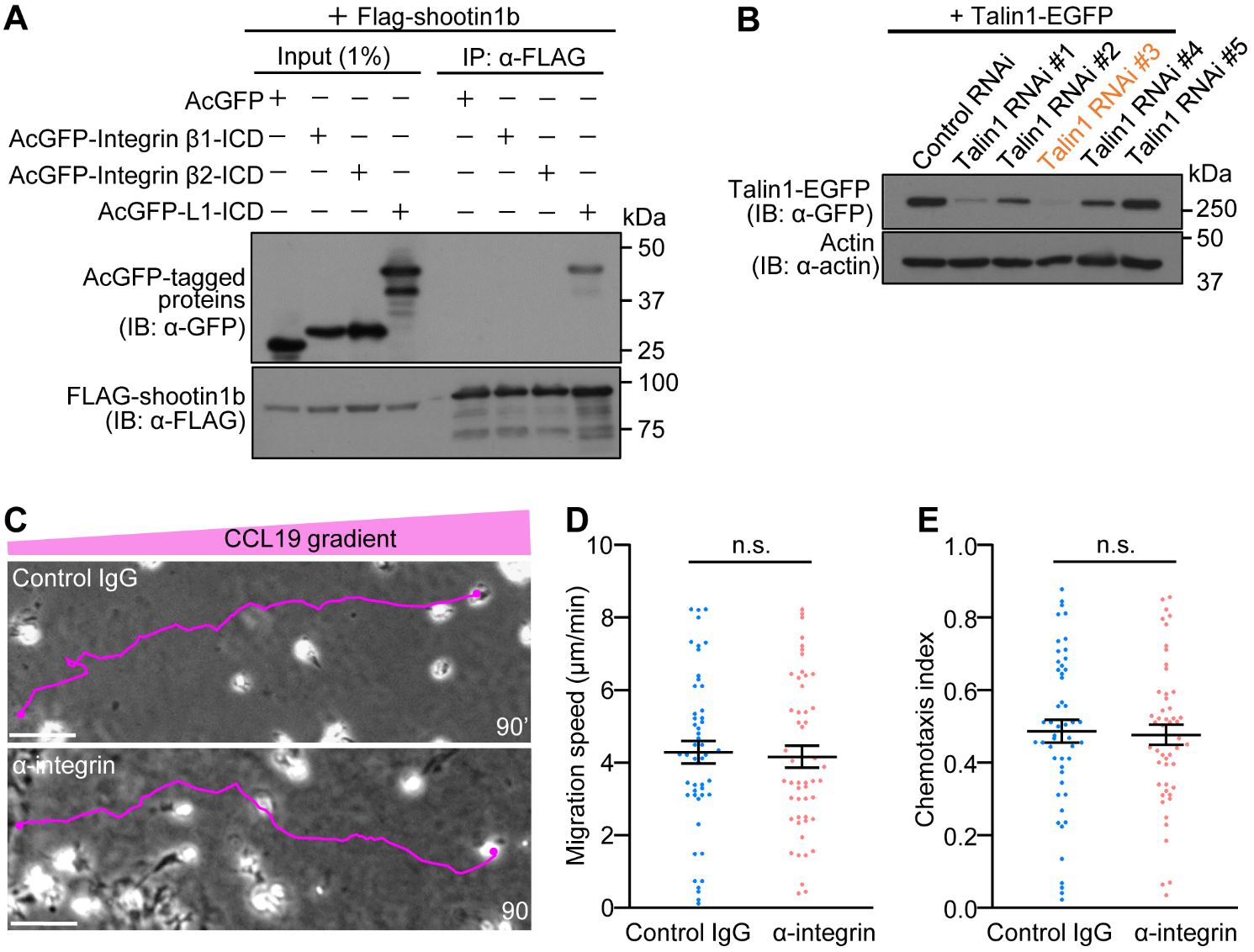


**Figure S7.** Shootin1b does not interact with β1- or β2-integrin. A) Co-immunoprecipitation assay of AcGFP (control), AcGFP-integrin β1-ICD (intracellular domain), AcGFP-integrin β2-ICD and AcGFP-L1-ICD with flag-shootin1b. Cell lysates from HEK293T cells, expressing AcGFP-tagged proteins and flag-shootin1b, were incubated with anti-flag antibody. The immunoprecipitates and cell lysates (1 %) were immunoblotted with anti-GFP or anti-flag antibody. AcGFP-L1-ICD, but not AcGFP-integrin β1-ICD or AcGFP-integrin β2-ICD, was co-precipitated with flag-shootin1b. B) HEK293T cells were co-transfected with mouse talin1-EGFP vector and candidate mouse talin1 RNAi vectors (#1 ~ #5). Cell lysates were then analyzed by immunoblot with anti-GFP. Actin was used as a loading control. Talin1 RNAi vector #3 was used in the knockdown analysis. C) Dendritic cells were cultured in a mixture of collagen gel and Matrigel with control IgG (6 µg/mL) or α-integrin β2 (6 µg/mL) for 1h in the absence of CCL19 gradient. One hour after application of the CCL19 gradient, time-lapse phase-contrast images of dendritic cells were obtained. Nuclei were also visualized by Hoechst to trace the trajectories of cell migrations. The pictures show representative images from the time-lapse series taken every 1 min for 90 min. Tracing line (magenta) indicate dendritic cell migration for 90 min. Scale bar: 50 μm. D, E) Analyses of the migration speed (D) and chemotaxis index (E) of dendritic cells in (C). Two-tailed unpaired Student’s *t*-test was performed (control IgG, n = 50 cells; α-integrin β2, n = 50 cells). Data represent means ± SEM; ns, not significant.


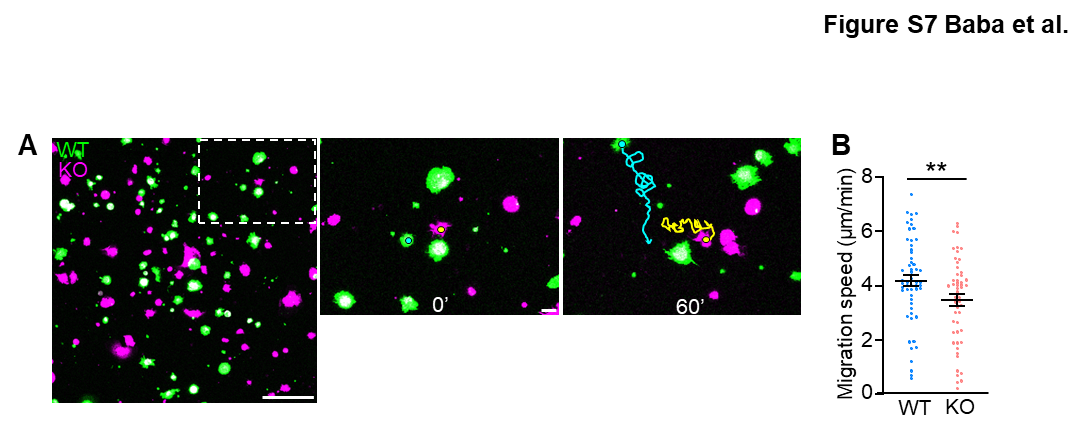


**Figure S8.** Shootin1b KO inhibits dendritic cell migration in lymph node slice. A) Fluorescence images of WT (green) or shootin1b KO (magenta) cells migrating in a lymph node slice. Enlarged images in the rectangle area are shown to the right; the pictures show representative time-lapse images from the time-lapse series taken every 30 sec for 60 min. See Video S10. Scale bar: 50 µm (in the inset, 20 µm). B) Analyses of the migration speeds of WT and shootin1b KO dendritic cells in lymph node slices in (A). Two-tailed unpaired Student’s *t*-test was performed (WT, n = 56 cells; KO, n = 55 cells). Data represent means ± SEM; **, p < 0.02.


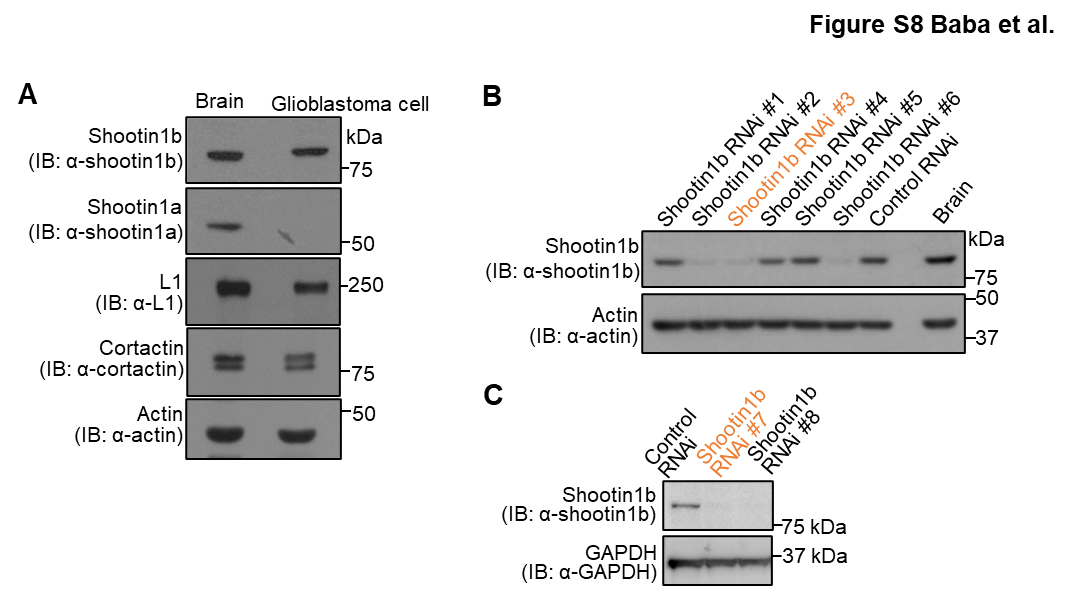


**Figure S9.** Expression of shootin1b, cortactin and L1 in human glioblastoma cells. A) Immunoblot analysis of human glioblastoma cells with anti-shootin1b, anti-shootin1a, anti-L1 and anti-cortactin antibodies. Actin was used as a loading control. Embryonic day 16.5 mouse brain lysate was used as positive control for the detection of endogenous shootin1a, shootin1b, L1 and cortactin. B) HEK293T cells was transfected with human shootin1b RNAi candidate vectors (#1 ~ #6). Cell lysates were then analyzed by immunoblot with anti-shootin1b. Actin was used as a loading control. We used shootin1b RNAi vector #3 to knockdown shootin1b in glioblastoma cells in Figures 6F, H-I, 7B-C. C) Human glioblastoma cells were infected with lentivirus carrying shScramble (control RNAi) or candidates of shShootin1b (shootin1b RNAi #7, #8). Glyceraldehyde-3-phosphate dehydrogenase (GAPDH) was used as a loading control. We used shootin1b RNAi vector #7 to knockdown shootin1b in glioblastoma cells in Figure 7H-I.


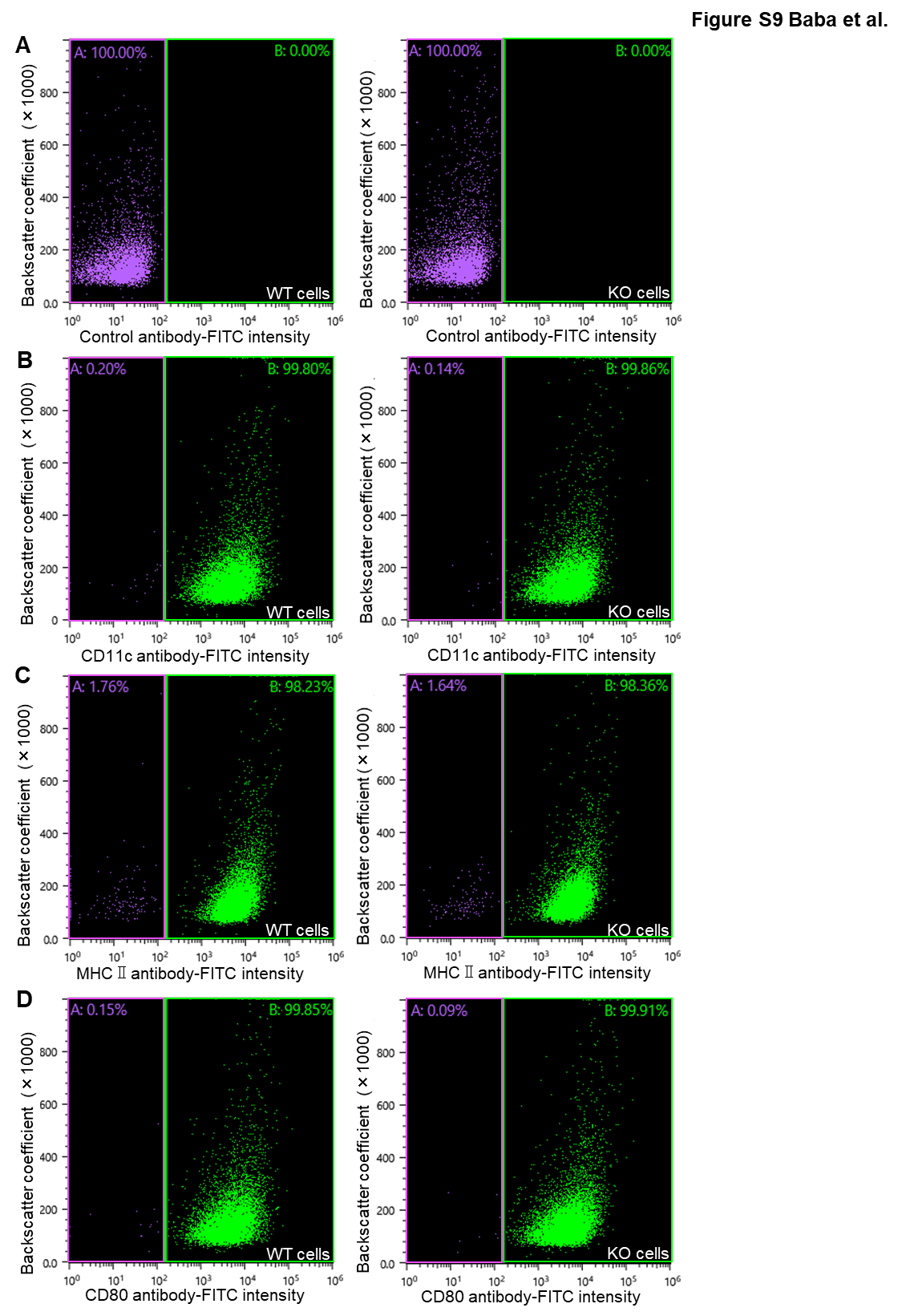


**Figure S****10.** Shootin1b KO does not affect dendritic cell maturation. A-D) Flow cytometry analysis of the expression of dendritic cell surface markers on WT and shootin1b KO dendritic cells after maturation. Dendritic cell surface markers against CD11c (B), MHC-Ⅱ (C), CD80 (D) were detected with FITC-conjugated antibodies. The corresponding isotype control antibody did not bind to dendritic cell surface (A). Dendritic cell population was detected as dot plot. The x and y axis of graphs indicate FITC intensity and backscatter coefficient, respectively. Magenta rectangles in the graphs indicate FITC-negative cell population (A). Green rectangles indicate FITC-positive cell population (B-D). Numbers within magenta and green rectangles represent the percentage of FITC-negative and FITC-positive cell population to the total cell number, respectively.

・Preparation of 1.5 mg /mL collagen gel + 10 % matrigel for chemotaxis assay

**Supplementary Table S1**


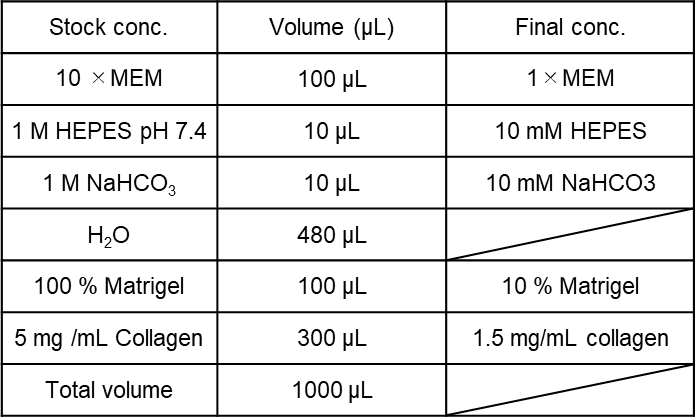


・Preparation of 1.5 mg /mL collagen gel + 10 % matrigel + CCL19 (20 or 200 ng/mL) for


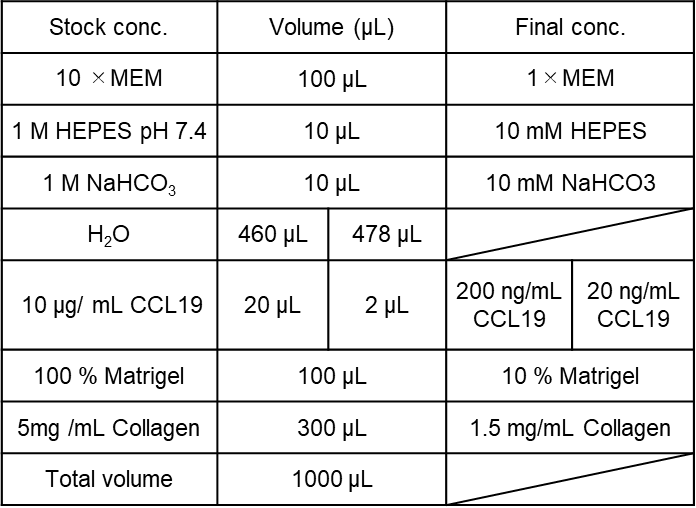
random migration assay in the bath application of CCL19

・10 ×MEM (Sigma, catalog number: M0275)

・1M HEPES, pH 7.4 (Nacalai, catalog number: 17557-94)

・Matrigel (Corning, 356231)

・5 mg/mL Collagen (IAC-50, catalog number: IAC-50)

・CCL19 (R&D system, catalog number: 440-M3-025)

**Table S1. Preparation of collagen gel + matrigel mixture for dendritic cell 3D migration.**

**Supplementary Video legends**

**Video S1. A time-lapse fluorescence movie of traction forces exerted by a dendritic cell migrating in the presence of 200 ng/mL CCL19.** Blue lines indicate the movement of the beads from their original positions. Yellow arrows indicate the direction and relative amplitude of traction forces. See the legend for Figure 1B.

**Video S2. Time-lapse fluorescence movies of dendritic cells migrating in collagen gel + Matrigel in the presence of 20 and 200 ng/mL CCL19.** Nuclei were visualized by Hoechst to accurately trace the trajectories of cell migrations. See the legend for Figure S1A-C.

**Video S3. A time-lapse fluorescence movie of traction forces exerted by WT and shootin1b KO dendritic cells migrating in the presence of 200 ng/mL CCL19.** See the legends for Figure 1B, G.

**Video S4. A time-lapse fluorescence movie of traction forces exerted by dendritic cells overexpressing EGFP (control) or EGFP-shootin1-DN and migrating in the presence of 200 ng/mL CCL19.** See the legends for Figure S3A-B.

**Video S5. Time-lapse fluorescence movies of WT and shootin1b KO dendritic cells migrating in collagen gel + Matrigel in the presence of 200 ng/mL CCL19.** Nuclei were visualized by Hoechst to trace the trajectories of cell migrations. See the legend for Figure S1A, D.

**Video S6.** **Movement of fluorescent speckles of HaloTag-actin, HaloTag-shootin1b and L1-halotag at the leading edge of dendritic cells on laminin (LN)- or PDL-coated glass.** See the legend for Figure 2A-C, F-H.

**Video S7.** **Time-lapse fluorescence movies of HaloTag-actin and Lifeact-EGFP at the leading edge of WT and shootin1b KO dendritic cells.** See the legend for Figure 3E.

**Video S8. Time-lapse fluorescence movies of WT and shootin1b KO dendritic cells, WT dendritic cells overexpressing EGFP (control) or EGFP-shootin1-DN under CCL19 gradient.** Nuclei were visualized by Hoechst to trace the trajectories of cell migrations. See the legend for Figures 4A-B, S6A-B.

**Video S9. Time-lapse fluorescence movies of WT and shootin1b KO dendritic cells expressing control RNAi or talin1 RNAi under CCL19 gradient.** Nuclei were visualized by Hoechst to trace the trajectories of cell migrations. See the legend for Figure 5D.

**Video S10. A time-lapse fluorescence movie of WT and shootin1b KO dendritic cells migrating in a lymph node slice.** See the legend for Figure S8A.

**Video S11. Time-lapse movies of migration of a human astrocyte and a glioblastoma cell, glioblastoma cells expressing control RNAi or shootin1b RNAi, and glioblastoma cells overexpressing EGFP (control) or EGFP-shootin1-DN.** See the legend for Figures 6C, 7A-B, D-E.

**Video S12.** **Movement of fluorescent speckles of HaloTag-actin at the tip of the tumor microtube of glioblastoma cells expressing control RNAi and shootin1b RNAi.** See the legend for Figure 6E.

**Video S13. A time-lapse fluorescence movie of traction force at the tip of the tumor microtube of glioblastoma cells expressing control RNAi and shootin1b RNAi.** See the legend for Figure 6G-H.

**References**

1. Timpl, R., Rohde, H., Robey, P.G., Rennard, S.I., Foidart, J.M., and Martin, G.R. (1979). Laminin--a glycoprotein from basement membranes. J. Biol. Chem. *254*, 9933-9937.
